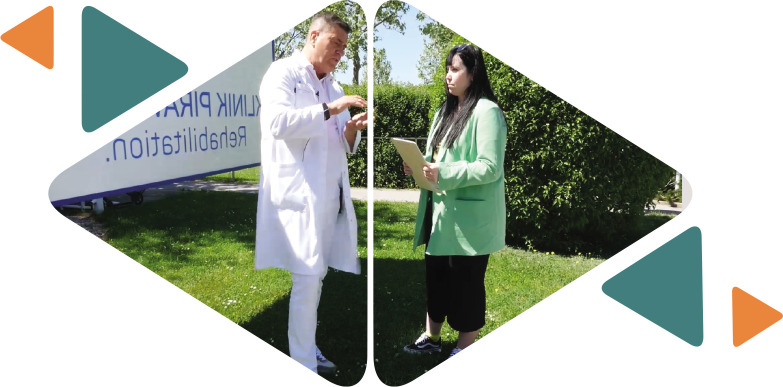# Andreas Winkler – Adapted interviews from Neurotrauma Treatment Simulation Center (NTSC) – Vienna, 2022

**DOI:** 10.25122/jml-2022-1021

**Published:** 2022-07

**Authors:** Andreea Strilciuc, Diana Chira, Alexandra-Mihaela Gherman

**Affiliations:** 1. RoNeuro Institute for Neurological Research and Diagnostic, Cluj-Napoca, Romania

**Interviewee: Andreas WINKLER**

**Interviewer: Diana CHIRA**

**D.C**.: Hello, Professor Winkler. First of all, thank you for having us here, at PIRAWARTH. [...] Can you tell us a few words about yourself, like your focus area in research and practice?

**A.W**.: Thank you, Diana. Klinik Pirawarth [...], the place where we are, is Austria's biggest neurorehabilitation centre, with about 200 beds for patients with neurological problems. I am a neurologist, geriatrician and rehab specialist and I am working here for decades our main focus is the recovery of lost functions. We try to improve motor functions, sensory functions, and neuropsychological functions in rehabilitation with a whole team of experts and with all available devices and methods that are evidence-based in medicine.

**D.C**.: That sounds really interesting. And what was your motivation for joining the coordination team for this program?

**A.W**.: Well, I think traumatic brain injury is still a challenge, even in Austria we have a good health care system, but in terms of TBI we have a long way to go, still to go, and I think the networking part of different countries, with our Austrian colleagues here, could be an impulse to improve and push the boundaries that restrict us in supplying the best care we could for our patients.

**D.C**.: Right. Could you describe how your experience shaped your opinion regarding the rehabilitation of neurotrauma? [...] Your whole experience, did it change the way your view neurotrauma and the rehabilitation process?

**A.W**.: Yes. I think the main points we have to take into account first are that rehabilitation would have to start very early, we should not waste time, [...], especially for TBI. The second thing is that we have to work together, all the disciplines in the neurorehabilitation process, with all our colleagues from neurosurgery, internal medicine, and psychiatry, we have to work together for the best outcome and we have to create a solid base of evidence. We do not have the same evidence amount of studies in the field of TBI science as, for example, in stroke medicine. So, there's a huge effort that we need more data, better data, and more studies, especially for TBI rehabilitation patients.

**D.C**.: That's correct. And about our program, are you familiar with similar programs to this one, and are you involved in any of them?

**A.W**.: Well, there was a similar program for stroke patients, but not for TBI, it was the AVANT program, perhaps you know that program when we tried to deal with the focus of stroke. But for TBI, this is [...] a flagship program, and I am very proud to be part of this program.

**D.C**.: Yes, we are too. If you had to identify some challenges in the approach of TBI, what would they be? Just a few of them, if you can mention them.

**A.W**.: Yes. Well, I think, of course, obviously there is prevention. Prevention, especially when so many elderly patients suffer from TBI because of falls, I think there is so much to do in the prevention of TBI, especially in elderly patients. The second is, if you already suffer from a TBI, the time from the acute hospital to the rehabilitation ward must be shortened, it must be very quick and most of our patients wait weeks for a place for rehabilitation. I think these have to change. And the third is we need funding. Because TBI is not only restoring some functions, motor functions, walking functions, and neuropsychological functions. It affects the patient in his whole surrounding, his calculus, he needs financial and moral support. I think there's still a way to go.

**D.C**.: So, what organizational issues do you think would be most pressing when it comes to the rehabilitation process for the patient?

**Figure:**
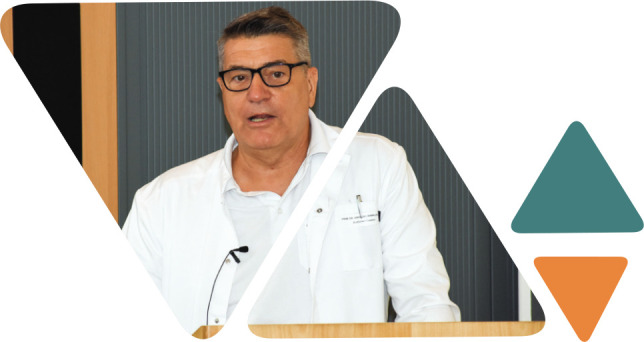


**A.W**.: I think we need better communication between acute centres, neurosurgery centres, pre-hospital management and rehabilitation centres. We have to connect these areas much better and, of course, on top of all these, what we need is a registry. We need to know what we do - if it is useful and if we can improve the outcome.

We don't know yet, we need more data. I think we need a registry, especially so that all the participants in this management circle of TBI can come together, work together, define the problems and solve them.

**D.C**.: Right. From your point of view, which are the main limitations in the approach to cognitive, behavioural or depressive disorders that result from neurotrauma?

**A.W**.: Well, it depends always individually, on the extent of these problems, but one thing is that we need time. The time...

First, we need to start early, the second is that we need time for these patients, they do not heal in 4 weeks or 6 weeks, sometimes it is a lifelong problem, so rehabilitation becomes a lifelong problem. [...] We should not forget that we have secondary, and tertiary problems we have to take into account. So, time and financial support are very important, so that affected patients with these severe handicaps have the possibility for a longer time period to profit from rehabilitation measures, in different kind of ways.

**D.C**.: Could you maybe describe, shortly, one of the most intriguing cases you've ever encountered? [...]

**A.W**.: Yes, you know, my special interest also is in problems with consciousness and I deal a lot of time with patients either in a vegetative state or regaining consciousness to the minim conscious state, and I remember a patient being vegetative for 5 years, for 5 years and then restarted special treatment with stimulation therapy, Snoezelen therapy and this patient regained, after 5 years, consciousness. Of course, she was severely handicapped, but this tells me never to give up on a patient [...].

**D.C**.: [...] Yes, it gives us hope. These days, online medical communities are becoming more and more visible. Considering this, what do you think is the role of an online community in medical practice today?

**A.W**.: I think this is important because it is a platform for us. We need to communicate and we need to exchange, we have to have a platform for organizing and planning our approaches, scientific approaches. Communication is very important, [...] this is the right platform for it.

**D.C**.: And how about the pandemic that we know, we all had to fight - how do you consider that COVID-19 has impacted the management or the rehabilitation process for the neurotrauma patients?

**A.W**.: Well, COVID affected the whole world and also, of course, rehabilitation facilities, but I think, in the core, we did the same as before the pandemic. I think the circumstances are harder, the restrainments are much bigger and the burden for the therapists and nurses and doctors has increased much. So, we are really, really exhausted. After nearly two and a half years of the pandemic, it's very hard for us to work at the same level as before. But you know, we are used to it, we are happy that we do not have so many cases now and we are hopeful that this pandemic will resolve and will not put too much burden on the EU (Emergency Unit) again. Because all the therapists and nurses and doctors are really exhausted by now.

**D.C**.: I can imagine it must be really hard. And for our last question, from your perspective, which are the obstacles that would affect a proper long-term follow-up for patients?

**A.W**.: I think this is a very good question because it is so important [...] that we shouldn't lose our patients in the long-term, we should implement a registry that we know exactly where the patient is, at which time, where are the controls, how we can find out how he proceeds, how he improves.

So, I think we have to make a commitment that we need a management cycle, with a registry, to follow our patients at least when they are stable, so we can say how can we improve, but we shouldn't lose our patients on the way. Because otherwise, we cannot learn from what we are doing right now. We have to improve.

**D.C**.: Definitely. Well, thank you for your time [...] and for your answers, and we are looking forward to the rest of the program today.

**A.W**.: Thank you very much!

Watch the extended interview on the AMN Website: https://brain-amn.org/ntsc-interviews-andreas-winkler/

**Figure:**